# Amyotrophic lateral sclerosis alters the metabolic aging profile in patient derived fibroblasts

**DOI:** 10.1016/j.neurobiolaging.2021.04.013

**Published:** 2021-09

**Authors:** Margarita Gerou, Benjamin Hall, Ryan Woof, Jessica Allsop, Stephen J. Kolb, Kathrin Meyer, Pamela J. Shaw, Scott P. Allen

**Affiliations:** a Department of Neuroscience, Sheffield Institute for Translational Neuroscience (SITraN), University of Sheffield, Sheffield, UK; b Department of Neurology, The Ohio State University Wexner Medical Centre, Columbus, OH, USA; c Centre for Gene Therapy, Nationwide Children’s Hospital, Columbus, OH, USA

**Keywords:** Aging, ALS, Metabolism, Fibroblasts, AGEs, advanced glycation end products, ALS, amyotrophic lateral sclerosis, AMPK, AMP-activated protein kinase, ChREBP, carbohydrate response element binding protein, C9ORF72, chromosome 9 open reading frame 72, DHAP, dihydroxyacetone phosphate, ETC, electron transport chain, FALS, familial amyotrophic lateral sclerosis, G3P, glyceraldehyde 3-phosphate, GP, glycogen phosphorylase, iAstrocytes, induced astrocytes, iNPC, induced neuronal progenitor cell, iPSC, induced pluripotent stem cells, LDHA, lactate dehydrogenase A, mtDNA, mitochondrial DNA, NADH, nicotinamide adenine dinucleotide, reduced form, PGM, phosphoglucomutase, SALS, sporadic amyotrophic lateral sclerosis, SOD1, superoxide dismutase 1, TCA, tricarboxylic acid, TDP-43, TAR DNA-binding protein 43, ROS, reactive oxygen species

## Abstract

Aging is a major risk factor for neurodegenerative diseases, including amyotrophic lateral sclerosis (ALS). As metabolic alterations are a hallmark of aging and have previously been observed in ALS, it is important to examine the effect of aging in the context of ALS metabolic function. Here, using a newly established phenotypic metabolic approach, we examined the effect of aging on the metabolic profile of fibroblasts derived from ALS cases compared to controls. We found that ALS fibroblasts have an altered metabolic profile, which is influenced by age. In control cases, we found significant increases with age in NADH metabolism in the presence of several metabolites including lactic acid, trehalose, uridine and fructose, which was not recapitulated in ALS cases. Conversely, we found a reduction of NADH metabolism with age of biopsy, age of onset and age of death in the presence of glycogen in the ALS cohort. Furthermore, we found that NADH production correlated with disease progression rates in relation to a number of metabolites including inosine and α-ketoglutaric acid. Inosine or α-ketoglutaric acid supplementation in ALS fibroblasts was bioenergetically favourable. Overall, we found aging related defects in energy substrates that feed carbon into glycolysis at various points as well as the tricarboxylic acid (TCA) cycle in ALS fibroblasts, which was validated in induced neuronal progenitor cell derived iAstrocytes. Our results suggest that supplementing those pathways may protect against age related metabolic dysfunction in ALS.

## Highlights

• Aging affects the metabolic profile of fibroblasts derived from ALS cases
• Increased NADH metabolism with age is observed in the presence of a specific set of catabolic energy substrates in healthy individuals
• Reduced NADH metabolism with age is observed in the presence of glycogen in the ALS cohort
• Disease progression rates in ALS cases correlate with fibroblast NADH production in the presence of a number of energy substrates including inosine

## 1 Introduction

Aging is considered one of the major risk factors for several neurodegenerative diseases including amyotrophic lateral sclerosis (ALS) and as a natural process is affected by several key mechanisms including metabolic alterations (Lopez-Otin et al., 2016). Mitochondria undergo significant damage during the aging process leading to progressive dysfunction and, together with alterations in intercellular communication; these factors play a crucial role in aging (Gonzalez-Freire et al., 2015). Studies investigating the relationship between mitochondrial DNA (mtDNA) and aging have shown an increase in germline and somatic mutations of mtDNA in mouse models of premature aging (Edgar et al., 2009; Trifunovic et al., 2004) as well as in aged humans (Ma et al., 2018). Moreover, age-related mitochondrial damage has been linked to increased reactive oxygen species (ROS) production (Ferrucci and Fabbri, 2018). Deficiencies in mitochondrial respiratory complexes have been found with aging causing an electron leak via an increase of the electron transport chain (ETC) redox state, which contributes to ROS overproduction (Golpich et al., 2017; Stefanatos and Sanz, 2018).

In addition to mitochondrial dysfunction, multiple studies have found differences in the levels of specific metabolites between young and aged individuals. This is observed in several species, including worms (Copes et al., 2015), flies (Hoffman et al., 2014), mice (Calvani et al., 2014) and humans (Chaleckis et al., 2016; Lawton et al., 2008). In humans, correlation of the metabolic phenotype of human red blood cells between aged and young people has shown reduced levels of metabolites associated with antioxidants, redox metabolism and muscle reinforcement in healthy elderly individuals (Chaleckis et al., 2016). Many of the metabolic abnormalities observed in natural aging are present in cellular, animal and patient derived models of ALS (Vandoorne, Tijs et al., 2018). The disease is characterised by degeneration of upper and lower motor neurons leading to death, primarily by respiratory muscle failure (Foster and Salajegheh, 2019). Although the majority of cases are classed as sporadic (SALS), around 10% of the patients are familial (FALS), usually with autosomal dominant inheritance (Ajroud-Driss and Siddique, 2015). The frequency of ALS increases with age, the majority of ALS patients are diagnosed between 50–75 years old and average disease duration post-diagnosis is 2–3 years (Wang et al., 2017). Several studies have shown that metabolic dysfunction is a key pathogenic mechanism in ALS which may influence the rate of disease progression (De Vos and Hafezparast, 2017; Haeusler et al., 2016; Tefera and Borges, 2016; Vandoorne, T. et al., 2018). Hypermetabolism, related to greater loss of motor neurons and faster disease progression has been observed in ALS patients (Steyn et al., 2018). A common factor in sporadic neurodegenerative diseases such as ALS is the functional deterioration of the ETC (Lin and Beal, 2006). Impairment of the function of the ETC and modification of gene expression related to the ETC has been observed in ALS models (Ferraiuolo et al., 2007).

Increased metabolic flexibility may be crucial in counteracting the bioenergetic deficit observed in patient and animal models of disease (Allen et al., 2019b). With this in mind, upregulation of glucose, fatty acid and amino acid pathways identified in ALS mouse models, has recently been proposed as a compensatory mechanism for energy defects (Szelechowski et al., 2018). More recently, *in vivo* experiments in a drosophila model of TDP-43 proteinopathy have shown increased glucose uptake and upregulation of glycolysis in patient derived induced pluripotent stem cell (iPSC) motor neurons, supporting a potential glycolytic neuroprotective role (Manzo et al., 2019). Mechanistic understanding of the role of ALS in human cellular metabolic catabolism would allow for the identification of pathways that could be nutritionally supplemented to support energy production with the potential to influence disease progression rates.

Human fibroblasts can be used as translational model to investigate ALS as they offer the genetic background of the patient and in many cases recapitulate the metabolic dysfunction observed in the CNS, as well as showing an altered metabolic response to aging (Allen et al., 2015; Allen et al., 2019a; Raman et al., 2015). Previous studies from our laboratory and others in fibroblasts isolated from SOD1 FALS cases, found decreased mitochondrial membrane potential, intracellular ROS elevation, decline of ATP production and upregulation of glycolysis (Allen et al., 2014; Liu et al., 2016). Furthermore, fibroblasts isolated from SALS cases exhibited a significant increase in glucose levels as well as hypermetabolism, probably as response to high ATP expenditure (Konrad et al., 2017). We have previously observed end-point changes in mitochondrial and glycolytic energy generation pathways in SALS fibroblasts compared to controls, which correlated with age (Allen et al., 2015). A limitation of this approach was that it did not evaluate the effect of age and ALS on the major catabolic pathways that feed into these energy-generating pathways, therefore limiting our understanding on the effect of ALS on aging in the context of metabolic dysfunction.

In two recent studies from our laboratory (Allen et al., 2019a; Allen et al., 2019b), we adapted a phenotypic metabolic approach that had previously been used to uncover tryptophan metabolic defects in autism patients (Boccuto et al., 2013). This methodology enables the comparison of healthy versus ALS cell models by simultaneously measuring energy production rates from 91 energy substrates, enabling a non-biased metabolic screen to be performed. The technology measures the ability of cells to produce NAD (P) H (nicotinamide adenine dinucleotides) in real time, via NADH producing catabolic pathways that utilise a range of metabolic substrates. The advantage of this approach is that is allows a live kinetic measurement of cellular bioenergetics. In contrast, measuring a cellular NADH/NAD+ ratio provides the redox state of the cell and is a measure of global cellular energy status (Cunnane et al., 2020), but does not necessarily pinpoint the upstream cause of dysfunction. However, our mechanistic approach in terms of aging and neurodegeneration is valid, as NADH and NAD+ are crucial cellular metabolic substrates/co-factors involved in multiple physiological pathways and extensive evidence links levels of these bioenergetic intermediates with CNS disorders and with aging (Covarrubias et al., 2021; Lautrup et al., 2019).

We used our novel approach to identify that fibroblasts from *C9orf72* and sporadic ALS cases have a distinct catabolic metabolic phenotype compared to healthy controls. Moreover, reprogramming these fibroblasts into induced neuronal progenitor cells (iNPC,) derived iAstrocytes (Meyer et al., 2014) leads to a loss of metabolic flexibility associated with impairment of nucleoside, glycogen, pyruvate and fructose metabolism.

Aging is well established as a risk factor for ALS and metabolic dysfunction is an early, significant pathophysiological mechanism. As the natural aging process also involves metabolic dysfunction and because dysregulation of energy metabolism may influence ALS disease progression, identifying how the metabolic aging process affects ALS is an important area of study. How aging affects the metabolic profile of fibroblasts and how this is affected by ALS has not previously been investigated. Therefore, we employed our novel metabolic profiling approach in fibroblasts isolated from FALS and SALS cases, correlated the data with age and compared the profiles to controls. In addition, we assessed whether energy production in the presence of each of the 91 metabolites correlated with ALS clinical parameters such as age of onset, age of death and disease progression rates. We validated our findings in iAstrocytes and fibroblasts using metabolic flux analysis and metabolic screening.

## 2 Methods

### 2.1 Human biosamples

Fibroblast samples were obtained from 15 age, sex matched controls, and 21 ALS cases, including six C9orf72 cases, five SOD1 cases, five TDP-43 cases and five sporadic cases (see Supplementary Table 1 and Table 2). The average age at the time of skin biopsy in controls and ALS fibroblast cases was 59 years (range 40–76 years) and 55 years (range 39–77 years), respectively. The average disease duration of the ALS cases was 42.9 (+/-24.6) months. iNPC samples were obtained from three controls and eight ALS cases including three *C9orf72* cases, three SALS cases and two SOD1 cases. The three SALS and two SOD1 cases were additional lines not included in the original fibroblast cohort of 36 samples. The average age at the time of skin biopsy in controls and ALS iNPC cases was 58 years (range 40-67 years) and 53 years (range 29–66 years), respectively. The average disease duration of the ALS cases was 70 (+/-61.5) months.

### 2.2 Fibroblast cultures

Skin biopsies were obtained from the forearm of subjects after informed consent and under sterile conditions, in accordance with guidelines set by the local ethics committee. Fibroblast cell cultures were established in supplemented cell culture medium (Lonza) with 10% foetal calf serum (Labtech), 2 mM glutamine, 50 mg/ml uridine, vitamins, amino acids and 1 mM sodium pyruvate in humid incubators at 37°C with 5% CO_2_.

### 2.3 iNPC culturing and iAstrocyte differentiation

iNPC culturing and iAstrocyte differentiation was performed as previously described (Allen et al., 2019a).

### 2.4 Ethical approval

Informed consent was obtained from all human subjects before skin sample collection (Study number STH16573, Research Ethics Committee reference 12/YH/0330).

### 2.5 Biolog phenotype microarray

#### 2.5.1 Preparation of fibroblast cultures

The preparation and phenotypic metabolic array analysis of the fibroblasts was performed as described previously (Allen et al., 2019a). Briefly, on day 1 Biolog PM-M1 plates, which contain different oxidisable carbon sources in each well, were coated with 30µl of IFM-1 (Biolog) inoculating fluid (containing 10% dialysed FBS and 0.3mM glutamine). The plates were then incubated overnight at 37°C/5% CO_2_ incubator. The next day, 96 well half-area plates were coated with 50µl of fibronectin (0.0025 mg/ml dilution in PBS). After one hour at room temperature, the plates were washed with 100µl PBS. Confluent fibroblasts were harvested by trypsinisation (Lonza) and the cell number was measured using a trypan blue dye exclusion test and a Countess automated cell counter (Invitrogen). PM-M1 plates incubated the previous day were used and the IFM-1 fluid containing the different metabolites was transferred to the corresponding wells on the fibronectin-coated plates. Next, 16,000 cells per well were resuspended in IFM-1 media, transferred to each well of the substrate plate and then incubated 37°C/5% CO_2_ for 40 h. After the stated incubation time, 10µl of redox dye mix MA (Biolog) was added to each well and the plates sealed with sterile Seal-Plate film to stop gas transfer. Dye colour change was measured every 20 min for 120 min and then every 60 min up to 300 min using a BMG Omega Pherastar at both 590 and 790nM or an OminiLog™ bioanalyser. After incubation, the plates were washed three times with 100µl of PBS and stored overnight at 80°C prior to cell counting. All results were normalized to cell number by addition of CyQUANT (Invitrogen) to each well as per the manufacturer’s instructions (1/400 dilution of the dye in HBSS buffer, 100µl per well) and fluorescence was measured using a BMG Omega Fluorostar. The dye colour change of TDP-43 patient-derived fibroblasts, *C9orf72* patient-derived fibroblasts and controls were measured using a BMG Pherastar plate reader. SOD1 patient-derived fibroblasts, SALS patient-derived fibroblasts and relevant control dye colour changes were measured using an OmniLog™ bioanalyser. Principle component analysis (PCA) plots at 300 minutes were generated using Qlucore Omics Explorer 3.6, with *p* < 0.05 taken as significant. Qlucore calculates eigenvectors (also known as principal components), which determine the directions of a feature in space. The eigenvalues determine the magnitude of separation and the variation of the data along axes. Qlucore orders the eigenvectors based on the amount of the total variance captured by each component, considering all variables or samples.

#### 2.5.2 Preparation of iAstrocyte cultures

iAstrocytes were cultured and analysed as previously described (Allen et al., 2019a; Allen et al., 2019b).

### 2.6 Starvation induced toxicity

Starvation induced toxicity was assessed based on cell numbers in the negative wells at the end of each assay, using CyQUANT analysis as previously described (Allen et al., 2019a; Allen et al., 2019b). Toxicity was calculated by assessing the level of cell survival in the negative wells compared to the glucose control at the end of each assay using the following equation:

$$\begin{array}{ccc} & & 100-((\text{CyQUANT}\;\text{value}\;\text{of}\;\text{negative}\;\text{well})/\\ & & \left(\text{average}\;\text{CyQUANT}\;\text{value}\;\text{of}\;\text{glucose}\;\text{wells})\times 100\right) \end{array}$$

### 2.7 Metabolic flux analysis

Mitochondrial and glycolytic stress test analysis in fibroblasts and iAstrocytes were performed on an XF24 bioanalyser as previously described (Allen et al., 2015; Allen et al., 2019a; Allen et al., 2014; Raman et al., 2015). Specifically for supplementation assays, iAstrocytes or fibroblast media was supplemented with 5mM glucose and 0.3mM glutamine in the absence or presence of either 5mM fructose, inosine or α-ketoglutaric acid for 24 hours prior to metabolic flux analysis. Flux analysis was performed using XF basal media (Agilent) supplemented as above. Metabolic flux analyses under non stress and stress conditions were assessed following sequential addition of the mitochondrial inhibitors oligomycin, FCCP and rotenone/antimycin (all from Sigma) as previously described (Allen et al., 2019a; Allen et al., 2014). Flux data were normalised to cell number using CyQUANT following the manufacturer’s instructions as previously described (Allen et al., 2019a).

### 2.8 Western blot analysis

Samples were analysed as described previously (Allen et al., 2019a; Allen et al., 2019b). Briefly, samples were lysed in RIPA buffer before being loaded onto a 10% SDS-PAGE Mini-PROTEAN Tetra Handcast systems (Bio-Rad). Proteins were resolved and transferred to a polyvinylidene difluoride membrane (PVDF, Millipore) at 250 mA for 1 h. The PVDF membranes were incubated for 1 h with blocking solution containing 5% BSA in Tris-buffered saline with 0.01% Tween (TBS-T). Subsequently, membranes were incubated overnight at 4°C with the following primary antibodies at 1:1000 dilution in blocking solution: rabbit actin (Abcam ab8227), rabbit glycogen phosphorylase (Proteintech 12075–1-AP) and rabbit phosphoglucomutase (Proteintech 15161–1-AP). Membranes were washed in TBS-T prior to incubation with HRP-linked rabbit secondary antibody at 1 in 5000 before detection by chemiluminescence (EZ-ECL HRP kit, Biological Industries) using a G:BOX (Syngene). Protein quantification levels were obtained by densitometry using GeneTools software (version 4.03.05, Syngene) normalised to the loading controls.

### 2.9 Statistical analysis

To overcome any potential data collection bias, all metabolic data were normalised to the internal glucose control and were analysed in at least triplicate and then correlated with age using Pearson’s correlation analysis using Graphpad Prism version 8.4.3. As each energy substrate produces a unique kinetic profile (Allen et al., 2019a; Allen et al., 2019b) controls were compared to FALS and SALS cases assessing the effect of age (age at biopsy) at two time points, 120 and 300 minutes to determine any significant aging effects. Metabolic data from ALS cases were also correlated with clinical parameters such as age of onset, age of death and disease progression. All metabolic flux data were assessed for Gaussian distribution prior to either unpaired t-test with a Welch correction or Mann-Whitney analysis. All graphs were generated showing standard deviation using Graphpad Prism version 8.4.3 (GraphPad Software, La Jolla, CA, USA).

## 3 Results

### 3.1 Young and old ALS fibroblasts show the greatest alteration in metabolic profile compared to controls

**Fig. 1:**
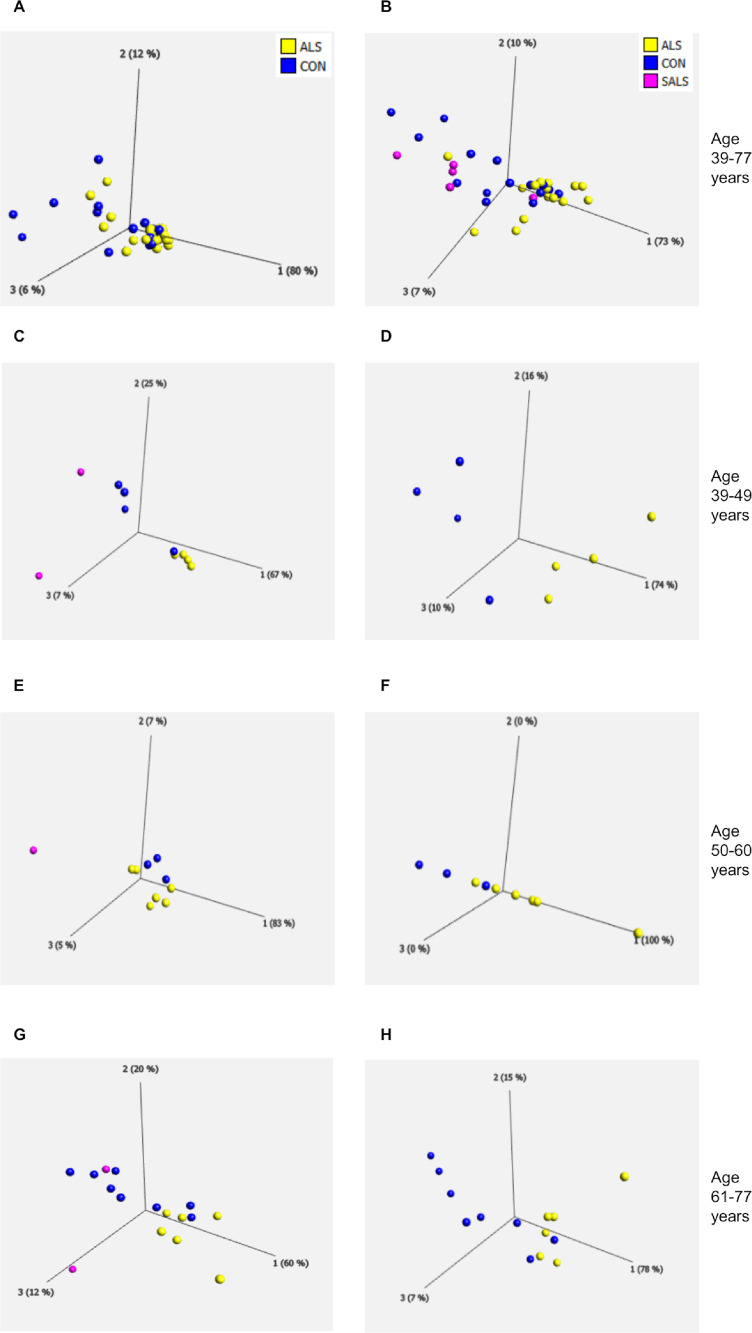
Age influences the metabolic profile of fibroblasts derived from *ALS* cases (A) PCA of control fibroblasts (blue, Con) and ALS fibroblasts (both FALS and SALS yellow) at the assay end point (300 minutes). (B) PCA of control fibroblasts (blue, Con) and SALS fibroblasts (pink) and FALS fibroblasts (yellow) at the assay end point (300 minutes). (C) PCA of control fibroblasts (blue, Con), SALS fibroblasts (pink) and FALS fibroblasts (yellow) under the age of 50 (39-49 years). (D) PCA of control fibroblasts (blue, Con) and FALS fibroblasts (yellow) under the age of 50 (39-49 years). (E) PCA of control fibroblasts (blue, Con), SALS fibroblasts (pink) and FALS fibroblasts (yellow) between 50-60 years. (F) PCA of control fibroblasts (blue, Con) and FALS fibroblasts (yellow) between 50 and 60 years, (G) PCA of control fibroblasts (blue, Con), SALS fibroblasts (pink) and FALS fibroblasts (yellow) 61-77 years. (H) PCA of control fibroblasts (blue, Con) and FALS fibroblasts (yellow) 61-77 years. Data presented as the mean of at least three biological replicates using 15 control fibroblasts, and 21 ALS fibroblasts. Analysis performed on Qlucore with the *p*-value set to ≤0.05. Q-values were 0.762 for control fibroblasts versus ALS (FALS+SALS) fibroblasts and 0.395 for control fibroblasts vs FALS fibroblasts. Percentage values represent eigenvectors calculated for each analysis. The higher the percentage the greater the confidence of the separation based on the vector. (Color version of figure is available online)

Aging is a naturally occurring process as well as one of the major ALS risk factors (Mattson and Arumugam, 2018). Therefore, we investigated the effect of age on the metabolic profile in fibroblasts from control and ALS cases using a novel phenotypic metabolic microarray approach (Allen et al., 2019a; Allen et al., 2019b). This methodology allows an unbiased screen of energy substrates that produce the reduced form of NADH. A single substrate as an energy source is contained in each well of a 96-well plate. Subsequently NADH production is monitored by addition of a proprietary dye that is reduced into a coloured product in the presence of NADH. To initially assess whether ALS alters the metabolome producing a distinct metabolic profile compared to controls, we performed PCA analysis on the data produced by the metabolic screen. Fig. 1A shows that, when taking all cases into account (15 controls and 21 ALS cases) there was distinct overlap between the two groups which still occurred if the ALS cases were split into FALS and SALS (Fig. 1B, FALS yellow, SALS pink). When we eliminated age from the analysis, we found all cases clustered at a single point (data not shown) indicating that age may play a significant role in defining the metabolic profile of the fibroblast cohort. To investigate this further we split our cohort into three age groups, 39–49, 50-60 and 61–77 years and performed the PCA analysis again on these distinct age groups (Fig. 1C-H). We found that if we compared SALS vs FALS vs controls (Fig. 1C, E, G) or if we just compared FALS vs controls (Fig. 1D, F, H) the greatest separation in the data sets occurred with the youngest and the oldest ALS cases. This PCA analysis indicated that in fibroblasts, ALS results in an altered metabolic profile, which is influenced by age.

### 3.2 Control fibroblasts increase metabolism of multiple energy substrates with age, which is not recapitulated in ALS fibroblasts

To assess which specific energy substrates lead to the altered metabolic profile in the fibroblasts with respect to age, we performed correlation analysis on all 91 substrates comparing NADH production with age. We have previously shown that ALS iAstrocytes (unlike ALS fibroblasts) have reduced metabolic flexibility and are more susceptible to starvation induced toxicity (Allen et al., 2019b). However, to check whether age affected the ability of fibroblasts to adapt to bioenergetic stress, which may influence our results, we measured cell numbers in the negative (absence of metabolite) wells on the phenotypic metabolic profiling plate after each assay. Neither control nor ALS fibroblasts showed any significant correlation between starvation induced toxicity levels and age (Supplementary Fig. 1). Moreover, we correlated the levels of specific toxicity of every metabolite on the phenotypic metabolic profiling plate individually with age and found no age-related effects (data not shown). Therefore, we were confident that any changes observed were not due to significant differences in cell survival with age within the groups.

**Fig. 2:**
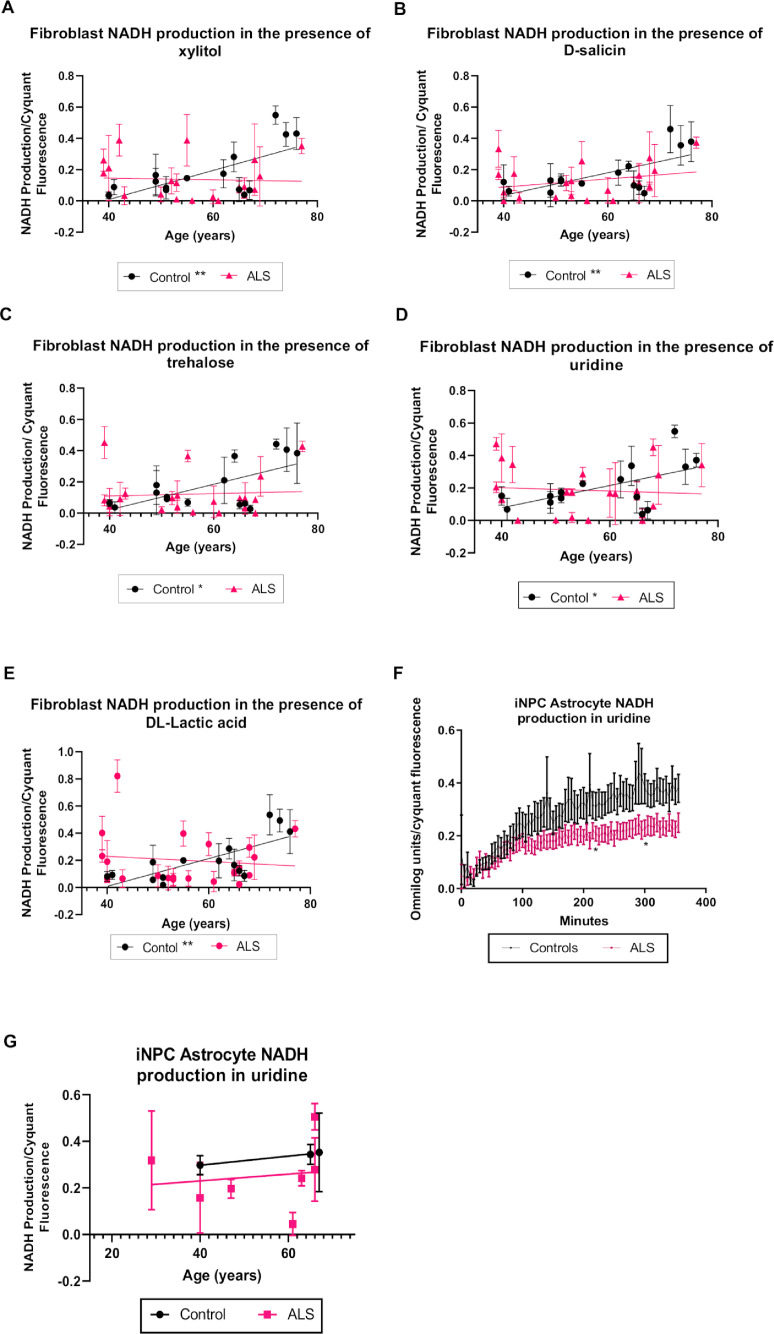
NADH production increases with age in control but not in ALS fibroblasts in the presence of xylitol, D-salicin, trehalose, uridine and lactic acid. (A) NADH production with age in the presence of xylitol. (B) NADH production with age in presence of D-salicin. (C) NADH production with age in presence of trehalose. (D) NADH production with age in presence of uridine. (E) NADH production with age in presence of DL-lactic acid. Data presented as mean with standard deviation of at least three biological repeats per cell line. Pearson’s correlation analysis was performed with the *p* value set to ≤ 0.05. Control fibroblasts (N=15, black), ALS fibroblasts (SALS and FALS *n*=21, pink). (F) NADH metabolism in the presence of uridine in iAstrocytes. Two-way annova with Sidaks’s post-test analysis was performed on three control lines and eight ALS lines performed in triplicate. Data presented as mean with standard error. (G) iAstrocyte NADH production with age in the presence of uridine. Data presented as mean with standard deviation *p ≤ 0.05, **p ≤ 0.01. (Color version of figure is available online)

Seven of the 91 metabolites showed a significant increase in NADH metabolism with age in control fibroblasts (Supplementary Table 3). Xylitol is a known sugar alcohol converted to xylose by a D-xylulose reductase, which is an intermediate of the pentose-phosphate pathway (Borgstrom et al., 2019). In the presence of xylitol, NADH production significantly increased with age in the control cohort (*p*=0.0066, *r*=0.6672), which was not observed in the ALS cohort (Fig. 2A). Metabolism of salicin, an alcoholic β-glucoside extracted from plants and broadly used as an analgesic and anti-inflammatory agent (Akao et al., 2002), also positively correlated with age in control fibroblasts (*p*=0.0073, *r*=0.6606). As with xylitol, no significant correlation was observed in the ALS cases (Fig. 2B). NADH production from trehalose, which is metabolised by the enzyme trehalase and rapidly hydrolysed to glucose (Lee et al., 2018), also showed a positive correlation with age in control fibroblasts (*p*=0.0106, *r*=0.6371). However, no significant correlation was observed between trehalose NADH production and age in ALS cases (Fig 2C). When uridine, a pyrimidine nucleoside linked to glycogen synthesis, lipid metabolism, and amino acid metabolism (Zhang et al., 2020) was provided as a sole energy source in fibroblast cultures, an increase in NADH production was observed with age in the control cases (*p*=0.0257, *r*=0.5726) but not in the ALS cases ALS (Fig. 2D). Similar results were observed in the presence of DL-lactic acid, which is converted to pyruvate by lactate dehydrogenase producing NADH as a biproduct (Ngo and Steyn, 2015) (Fig 2E, *p*= 0.0018,*r*= 0.7342). To validate these findings, we assessed NADH production in iNPC iAstrocytes reprogrammed from fibroblasts from ALS and control cases (Allen et al., 2019a; Allen et al., 2019b). Unlike fibroblasts, iAstrocytes did not metabolise salicin, xylitol or trehalose to a sufficient level to warrant metabolic kinetic analysis. We have previously published data showing a defect in lactic acid metabolism in iAstrocytes from SALS cases (Allen et al., 2019a). When assessing lactic acid metabolism in the remaining ALS cases (SOD1 and C9orf72), significant toxicity was observed in the ALS cases only, making analysis impossible and suggesting starvation induced toxicity due to an inability to metabolise lactic acid. Finally, we assessed uridine metabolism in our ALS iAstrocyte cases (Fig. 2F) and found a decrease in NADH production with this metabolic substrate, indicating a uridine metabolism defect in ALS cases as suggested by the fibroblast data. No age-related changes were observed with uridine metabolism (Fig. 2G) but due to low iAstrocyte numbers, our analysis was limited.

**Fig. 3:**
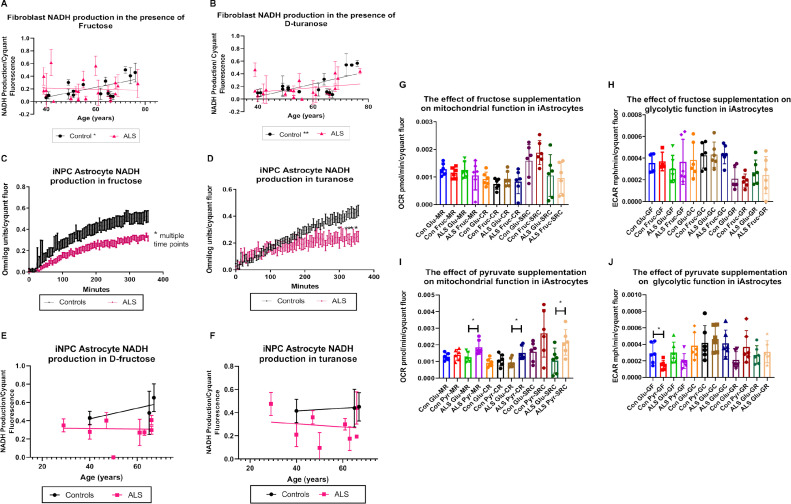
NADH production increases with age in control but not in ALS fibroblasts in the presence of fructose energy substrates. (A) NADH production in fibroblasts in the presence of fructose (B) NADH production in fibroblasts in presence of D-turanose. Controls (N=15) shown in black and ALS (SALS and FALS *n*=21) group showed in pink. Data presented as mean with standard deviation of at least three biological repeats per cell line. Pearson’s correlation analysis was performed with the *p* value set to ≤ 0.05. (C) NADH metabolism in the presence of fructose in iAstrocytes. (D) NADH metabolism in the presence of turanose in iAstrocytes. Two-way annova with Sidaks’s post-test analysis was performed on three control lines (black) and eight ALS lines (pink) performed in triplicate. Data presented as mean with standard error. (E) NADH production in fructose with age in iAstrocytes. (F) NADH production in turanose with age in iAstrocytes. Data presented as mean with standard deviation. (G) The effect of fructose supplementation on iAstrocyte mitochondrial function. (H) The effect of fructose supplementation on iAstrocyte glycolytic function. (I) The effect of pyruvate supplementation on iAstrocyte mitochondrial function. (J) The effect of pyruvate supplementation on iAstrocyte glycolytic function. (G-J) Data presented as mean with standard deviation of two control and two ALS cases performed in triplicate. Data was analysed using unpaired t-test analysis with a Welch correction. MR = mitochondrial respiration. CR = coupled respiration. SRC = spare respiratory capacity. GF = glycolytic flux. GC = glycolytic capacity. GR = glycolytic reserve. *p ≤ 0.05, **p ≤ 0.01. (Color version of figure is available online)

Interestingly, differences in NADH production between ALS and control cases were also observed upon exposure to two fructose based saccharides, fructose and D-turanose. The monosaccharide fructose, which is metabolised by fructokinase to generate fructose-1-phosphate (Levi and Werman, 1998), showed a significant positive correlation of NADH production with age in controls (*p*= 0.0166 and *r*= 0.6061) (Fig. 3A). Moreover, we found a significant increase in NADH production with age in the presence of the glycosylfructose energy substrate turanose. Turanose is hydrolysed to glucose and fructose by α-glucosidase, indicating a link between turanose and fructose metabolism (Julio-Gonzalez et al., 2021; Tewari and Goldberg, 1991; Zagalak and Curtius, 1975). Turanose metabolism increased in the control cohort with age (*p*=0.0072 and *r*=0.6755) (Fig. 3B). No significant correlation was observed between NADH production and age in the ALS cohort for either fructose or turanose. To validate these findings we assessed fructose and turanose metabolism in the iAstrocyte cohort. As observed in previous studies with *C9orf72* iAstrocytes (Allen et al., 2019a), we found a decrease in our iAstrocyte ALS cohort when we include SALS and SOD1 iAstrocytes in the analysis (Fig. 3C). We found similar results to a lesser extent when we assessed turanose metabolism in the same assay (Fig. 3D). We correlated our findings with age and found similar results to the patterns observed with fibroblasts but due to the limited numbers, no statistical significance was observed. We then assessed whether fructose supplementation would be beneficial metabolically in iAstrocytes. Unlike pyruvate supplementation, which increased mitochondrial rather than glycolytic function in ALS iAstrocytes, fructose supplementation had minimal effects on mitochondrial function or glycolytic function under physiological or stress conditions in controls and ALS cases (Fig. 3G-J).

### 3.3 Glycogen metabolism in ALS fibroblasts negatively correlates with age

**Fig. 4:**
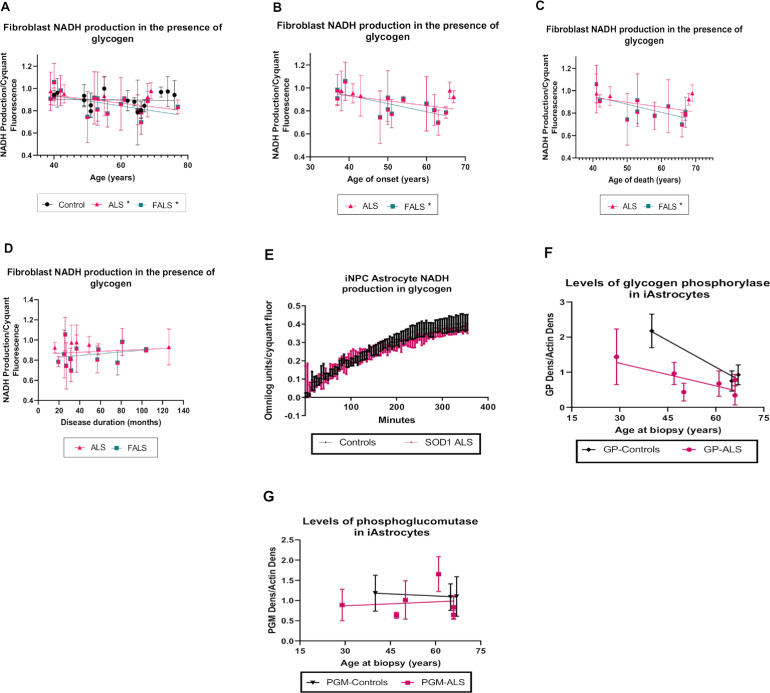
NADH metabolism in glycogen is reduced with age in ALS fibroblasts. (A) NADH production in fibroblasts in the presence of glycogen correlated with age of biopsy (B) NADH production in presence of glycogen as the sole energy source correlated with age of onset. (C) NADH production in presence of glycogen as the sole energy source correlated with age of death. (D) NADH production in presence of glycogen as the sole energy source correlated with disease duration. Data presented as mean with standard deviation of at least three biological repeats per cell line. Pearson’s correlation analysis was performed with the p value set to ≤ 0.05. Control fibroblasts (N=15, black), ALS fibroblasts (SALS and FALS, maximum *n*=21, pink, FALS only maximum *n*=16 blue). (E) NADH production in the presence of glycogen in SOD1 iAstrocytes. Data presented as mean with standard error 3 controls (black) vs 2 SOD1 cases (pink). (F) The effect of age on glycogen phosphorylase (GP) levels in iAstrocytes. (G) The effect of age on phosphoglucomutase (PGM) levels in iAstrocytes. Data presented as mean with standard deviation. *p ≤ 0.05. (Color version of figure is available online)

The polysaccharide glycogen is used as an energy source under several circumstances. For instance, in the CNS, glycogen is mainly stored in astrocytes and plays an important role as an energy fuel for motor neurons (Brown et al., 2003; Matsui et al., 2017). Glycogen is catabolized by glycogen phosphorylase (GP) and phosphoglucomutase (PGM) enzymes to generate glucose-6-phosphate, which we had previously shown were reduced in *C9orf72* iAstrocytes (Allen et al., 2019b). We found, in contrast with the previously identified alterations, that glycogen metabolism was reduced with age in the ALS cohort (*p*=0.0361, *r*=-0.4594) but not in control fibroblasts (Fig. 4A). This correlation was also observed when considering the familial ALS cohort alone (*p*=0.0217, *r*=-0.5680).

Based on the observed alterations in glycogen metabolism with age of biopsy in the ALS cohort (Fig. 4A), we hypothesised that glycogen metabolism may also negatively correlate with ALS clinical parameters such as age of onset, age of death or disease duration. In the ALS cases as a whole, although we observed a trend for decreased glycogen metabolism with age of onset and age of death this did not reach significance (*p*=0.055 and *p*=0.105 respectively). However, in familial cases, glycogen metabolism negatively correlated with age of onset and age of death (Fig. 4B, C, *p*=0.0138, *r*=-0.6858 and *p*=0.0307, *r*=-0.6794 respectively). No correlation was observed between glycogen metabolism and disease duration in fibroblasts (Fig. 4D). We have previously shown a reduction in glycogen metabolism in *C9orf72* iAstrocytes caused by loss of glycogen phosphorylase (GP) and phosphoglucomutase (PGM) (Allen et al., 2019a; Allen et al., 2019b). SALS cases showed increased heterogeneity in this regard, with only a subset matching the *C9orf72* data. When we analysed SOD1 iAstrocytes, we found no glycogen metabolism defect (Fig. 4E), suggesting perhaps that the glycogen metabolism changes observed in fibroblasts are driven by *C9orf72*. Based on our previously published data, we attempted to correlate glycogen metabolic enzymes with age in the *C9orf72* and SALS iAstrocytes (Fig. 4F-G and Supplementary Fig 2). Our analysis is limited by small numbers, however, we found a trend for decreased GP expression with age, which was exacerbated in ALS iAstrocytes (*p*= 0.061), and was not observed with PGM.

### 3.4 Metabolic correlations with ALS clinical parameters

**Fig. 5:**
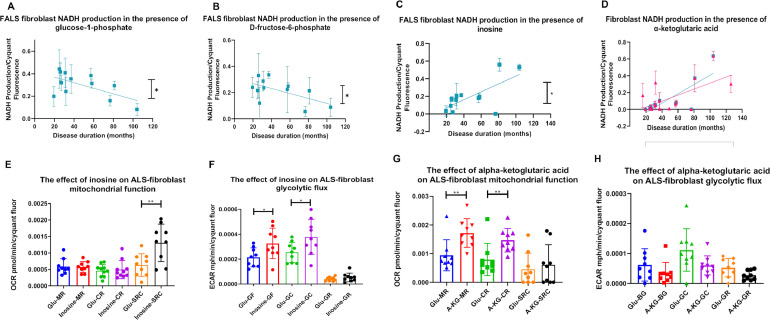
Disease progression length correlates with NADH metabolism in the presence of glucose-1-phosphate, D-fructose-6-phosphate, inosine and α-ketoglutaric-acid. (A) NADH production in FALS fibroblasts with glucose-1-phosphate as the sole energy source correlated with disease duration. (B) NADH production in FALS fibroblasts with D-fructose-6-phosphate as the sole energy source correlated with disease duration. (C) NADH production in FALS fibroblasts with inosine is the sole energy source correlated with disease duration. (D) NADH production with α-ketoglutaric-acid as the sole energy source correlated with disease duration. All data presented as mean with standard deviation of at least three biological repeats per cell line. Pearson’s correlation analysis was performed with the p value set to ≤ 0.05. ALS fibroblasts (*n*=17, pink), FALS fibroblasts (*n*=12, blue). (E) The effect of inosine supplementation on ALS fibroblast mitochondrial function. (F) The effect of inosine supplementation on ALS fibroblast glycolytic function. (G) The effect of α-ketoglutaric acid supplementation on ALS fibroblast mitochondrial function. (F) The effect of α-ketoglutaric acid supplementation on ALS fibroblast glycolytic function. Data presented as mean with standard deviation of three ALS cases performed in triplicate. MR = mitochondrial respiration. CR = coupled respiration. SRC = spare respiratory capacity. GF = glycolytic flux. GC = glycolytic capacity. GR = glycolytic reserve. Unpaired-test with Welch correction (E/F/H) or Mann-Whitney analysis (G) was performed on three ALS fibroblast cases in triplicate. Data presented as mean with standard deviation. *p ≤ 0.05, **p ≤ 0.01. (Color version of figure is available online)

Based on these data, we investigated whether clinical parameters were associated with changes in ALS fibroblast metabolic signatures. Therefore, the levels of NADH production from the 91 energy substrates, were correlated with ALS age of onset, age of death and disease duration. We identified four metabolites, where metabolism correlated with disease duration (Fig. 5). Metabolism of the glycolytic energy substrates glucose-1-phosphate and D-fructose-6-phoshate negatively correlated with disease duration in the familial cohort only (Fig. 5 A-B, *p*=0.0357, *r*=-0.6087 and *p*=0.0463, *r*=0.5838 respectively). No significant correlation was observed when incorporating the sporadic cases into the patient cohort (data not shown). The nucleoside inosine can be shuttled into the pentose phosphate pathway via ribose-1-phosphate and then into glycolysis, producing NADH, ATP and subsequently lactate (Balestri et al., 2007, Jurkowitz et al., 1998). Inosine metabolism positively correlated with disease progression in FALS cases (Fig. 5C, *p*=0.0146 and *r*=0.6819). Similarly, metabolism of the tricarboxylic acid (TCA) cycle substrate α-ketoglutaric acid positively correlated with disease duration in the ALS cohort as a whole (familial and sporadic) and in the FALS cohort alone (Fig. 5D, (*p*=0.0064, *r*=0.633 and *p*=0.0146, *r*=0.6819 respectively). To validate these findings, we assessed whether supplementation of inosine and α-ketoglutaric acid was metabolic beneficial to ALS fibroblasts. We found similar but distinct mechanisms of action between the two metabolic substrates (Figure 5E-H). Inosine as previously observed in ALS iAstrocytes (Allen et al., 2019a), increased mitochondrial and glycolytic capacity indicating a dual metabolic function (Fig. 5E-F). α-ketoglutaric acid increased fibroblast mitochondrial respiration as well as coupled respiration, indicating that supplementation induced a switch to a more aerobic metabolic profile (Fig. 5G). α-ketoglutaric acid had no significant effect on glycolytic flux (Fig. 5H). However, a trend for decreased glycolytic capacity (GC) and glycolytic reserve (GR) was observed (*p*=0.0704 and *p*=0.0513), confirming the switch to a more aerobic profile.

## 4 Discussion

Age related changes in bioenergetics of sporadic patient derived fibroblast as a model of ALS have been previously shown in our laboratory (Allen et al., 2015). Now using a novel phenotypic metabolic screening approach, we were able to assess in a larger cohort, the fibroblast metabolic profile with respect to aging and how that is affected by ALS. Our data suggest that fibroblasts from healthy controls display age related metabolic characteristics that are not recapitulated in fibroblasts from ALS cases. Several metabolites showed increased NADH production with age in fibroblasts derived from control cases, suggesting metabolic alterations in the energy source in these cells. Firstly, we found that xylitol showed significantly increased metabolism with age in control but not ALS cases (Fig. 2). A recent extensive review article summarizes the beneficial effect of xylitol on health by modulating immune function, intestine microbiota density, metabolic function and dental health amongst other benefits (Salli et al., 2019). Moreover, supplementation of xylitol has been shown to increase collagen synthesis in the skin of aged healthy mice indicating a potential protective effect (Mattila et al., 2005). At the metabolic level, xylitol is metabolised to xylulose-5-phosphate and activates the carbohydrate response element binding protein (ChREBP) via protein phosphatase 2A (Kabashima et al., 2003; Kawaguchi et al., 2001). When rats were provided with a high fat diet supplemented with xylitol, an increase of ChREBP and lipogenic enzymes was observed, suggesting a potential beneficial role of xylitol intake on obesity (Amo et al., 2011). To the best of our knowledge, xylitol has not been previously implicated in ALS. However, *in vivo* studies have shown an increase in lipid catabolism with a pre-symptomatic switch towards lipid oxidation in muscle being observed in SOD1^G86R^ mice (Palamiuc et al., 2015). Therefore, an inability to upregulate xylitol metabolism with age may support this lipid oxidation phenotype in ALS. With these data in mind, increasing xylitol metabolism in ALS may be protective.

An increase in trehalose metabolism was also observed with age in control but not in ALS fibroblasts (Fig. 2). Trehalose is a disaccharide broadly found in plants and bacteria as well as used as a nutritional supplement (Elbein et al., 2003). Previous *in vitro* evidence has suggested that trehalose can operate as an autophagy activator (Aguib et al., 2009; Casarejos et al., 2011; Sarkar et al., 2007). Several studies have also associated defects of autophagic function with aging and downregulation of autophagic genes in aged human brain (Plaza-Zabala et al., 2017). Although reduced autophagy has been observed in tissues with age, studies have shown that autophagy contributes to life extension (Madeo et al., 2015; Rubinsztein et al., 2011). Moreover, it now well established that the age-related decline in mitophagy (clearance of damaged mitochondria) is a contributing pathogenic factor in neurodegenerative disorders (Lou et al., 2020). Moreover, manipulating mitophagy levels decreases disease pathology and reverses cognitive decline (Fang et al., 2019). In the context of ALS, trehalose treatment delayed disease onset and induced longevity in SOD1^G93A^ mice via activation of mTOR-independent autophagy leading to protection of spinal cord motor neurons (Zhang et al., 2014). Delay of ALS disease progression was also shown in SOD1^G86R^ transgenic mice after administration of trehalose (Castillo et al., 2013). More recently, it was reported that trehalose was able to alleviate autophagic flux deficits in ALS through modifications of lysosomes (Rusmini et al., 2019). Therefore, the increase of trehalose metabolism in our control cohort with age may be a potential beneficial aging adaptation that is lost in ALS.

We also found that uridine showed significantly increased metabolism with age in control but not ALS fibroblast cases (Fig. 2D), with defects in uridine metabolism observed in ALS iAstrocytes (Fig. 2F). Uridine is a glycosylated uracil that plays a role in synthesis of glycolipids and glycoproteins, DNA and RNA (Deng et al., 2017). As a precursor of uridine triphosphate (UTP), uridine is able to stimulate the synthesis of glycogen (Mironova et al., 2018). Long term uridine supplementation has been shown to enhance lipid accumulation in the liver, and increase glucose levels in blood during fasting in mice (Urasaki et al., 2016). Moreover, uridine administration in wild-type mice under a high fat diet, as well as in an older cohort, improved glucose tolerance (Deng et al., 2017). Dose-dependent protective effects of uridine against sodium azide toxicity were observed in fibroblasts derived from Alzheimer’s disease patients (Garcia et al., 2005). In SOD1^G93A^ transgenic mice, uridine treatment slowed disease progression and increased motor performance probably by enhancing glycolytic energy production and increasing ATP (Amante et al., 2010; Ipata et al., 2010). In a recent metabolomic study, altered levels of nucleosides derivatives, including uridine-5′-monophosphate were found in fibroblasts from ALS cases (Veyrat-Durebex et al., 2019). Our data indicate that increased uridine metabolism with age might be beneficial for fibroblasts leading to the possibility of a protective role for uridine in ALS.

Another metabolite we found to have significantly increased metabolism with age in control but not ALS cases, was salicin (Fig. 2). Salicin is known for its analgesic and anti-inflammatory properties and is related chemically to salicylic acid and acetylsalicylic acid (Li et al., 2015; Zhai et al., 2018). *In vivo*, salicin is metabolised in the gastrointestinal tract to saligenin, which is converted by the liver to salicylic acid. *In vivo*, oral pre-treatment with salicin in a mouse model of rheumatoid arthritis reduced the inflammatory response through the NrF2-HO-1-ROS pathway (Zhai et al., 2018). Interestingly, salicin was able to modulate the expression of genes related to skin aging and a younger skin phenotype was generated (Gopaul et al., 2010). However, to the best of our knowledge, this is the first time that salicin metabolism has been linked with ALS.

Lactic acid metabolism was significantly increased with age in control fibroblasts, which was not observed in ALS cases (Fig. 2). These alterations were exacerbated in ALS iAstrocytes, which showed in general, starvation induced toxicity due to reduced lactate metabolism. Addition of lactate increased ATP production and cell survival in mice cortical neuronal cultures after glutamate injections to induce excitotoxicity, suggesting a neuroprotective role of lactate (Jourdain et al., 2016). Excitotoxicity is a process associated with aging as well as various pathologies, including ALS (Calvo et al., 2015). Evidence from aged mice suggests an increase of lactate levels in brain regions with physiological aging, as well as high mRNA levels of lactate dehydrogenase A (LDHA) (Ross et al., 2010). However, a decrease of LDHA protein level was associated with post-mitochondrial lactate elevation in the brain of aged mice, indicating that other mechanisms also play a role in lactate elevation (Datta and Chakrabarti, 2018). Defects in lactate metabolism have been linked to ALS metabolic dysfunction in previous studies (Ferraiuolo et al., 2011; Ngo and Steyn, 2015). Lactate levels in blood were age-dependent in SOD1^G93A^ transgenic mice, while low levels of lactate observed in striatum, cortex and brainstem of these mice correlated with disease progression (Lei et al., 2019).

Fructose and the fructose metabolic substrate turanose, which as with fructose can be used as an alternative sweetener (Park et al., 2016) showed increased metabolism with age in the control group, which was not recapitulated in the ALS cases (Fig. 3). Similar results were observed in iAstrocytes, which overall showed a decrease in fructose and turanose metabolism (Fig. 4C-E). Fructose is phosphorylated by fructokinase to generate fructose-one-phosphate, which is in turn metabolized to dihydroxyacetone phosphate (DHAP) and glyceraldehyde three-phosphate (G3P) and is able to enter the glycolytic pathway (Levi and Werman, 1998). Fructose supplementation in middle-aged rats enhanced the adverse effects of aging such ROS, inflammation and oxidative stress (Crescenzo et al., 2019; Harrell et al., 2018). A short-term fructose rich diet in adult rats has been shown to increase neuroinflammation via NF-κB in the hippocampus (Cigliano et al., 2018), with similar results being observed in the liver of middle-aged rats supplemented with high fat/fructose (Mazzoli et al., 2019). Unlike fructose, turanose may possess anti-inflammatory properties as IL-1β, IL-18, NOS and COX-2 levels were reduced in mouse macrophages in the presence of turanose (Chung et al., 2017). A fructose rich diet has been shown to correlate with increased fatty acid production and insulin resistance (Softic et al., 2017), which are both observed in older individuals (Toth and Tchernof, 2000). These data highlight the importance of controlling fructose consumption in healthy aging and suggest that the enhanced fructose metabolism with age we observed in our control fibroblast cohort may be in the physiological context, detrimental. It also raises the question of whether the lack of fructose metabolism with age observed in ALS cases may be a protective measure by the fibroblasts to reduce disease associated pathophysiological factors such as insulin resistance, ROS production and inflammation. High levels of pro-inflammatory markers, such as IL-6 and IL-8 have been observed in ALS patients (Blasco et al., 2017) and causative genes of ALS including *MATR3*, and *PFN1* and *TDP‐43* are enriched and associated with inflammation (Umoh et al., 2018). Moreover, several studies have demonstrated an increase in pro-inflammatory markers including IL-1, IL-6, TNF-α and C-reactive protein in healthy elderly individuals (Bruunsgaard et al., 1999; Franceschi and Campisi, 2014; Wei et al., 1992).

When we supplemented the iAstrocytes with fructose and measured metabolic flux, we observed no detrimental or restorative changes in mitochondrial respiration or glycolytic flux in ALS iAstrocytes (Fig. 3 G-H). However, when we supplemented with pyruvate, which unlike fructose enters the glycolytic pathway downstream, we found significant increases in mitochondrial function and a move towards an aerobic phenotype in the ALS iAstrocytes (Fig. 3 I-J). These data align with our previous findings (Allen et al., 2019a; Allen et al., 2019b) and suggest that pyruvate supplementation may be beneficial in ALS iAstrocytes and may circumvent the lack of lactic acid metabolism observed which causes starvation-induced toxicity in ALS cases. Fructose metabolism can lead to NADH production via production of three-phosphoglycerate. A reduction in NADH levels in response to enhanced exposure to fructose suggests that this step is not occurring efficiently which would be predicted to lead to an accumulation of the glycolytic intermediates glyceraldehyde-3-phosphate (GAP) as well as dihydroxyacetone phosphate (DHAP). Increased levels of both intermediates enhance the production of dicarbonyls such as methylglyoxal, which in turn lead to more stable advanced glycation end products (AGEs) (Hamada et al., 1996). In turn, AGEs disrupt many cell functions including lipid synthesis and antioxidant defences, leading to inflammation and mitochondrial metabolism (Aragno and Mastrocola, 2017). AGE receptors (RAGEs) have been found to be upregulated in spinal cord tissues isolated from ALS patients (Juranek et al., 2015) and SOD1 mice lacking RAGE exhibited slower disease progression (Lee et al., 2020). We have previously shown that fructose metabolism is reduced in fibroblasts and iAstrocytes from *C9orf72* ALS cases and that the glyoxalase enzymes required for MGO removal are reduced in *C9orf72* and SALS cases (Allen et al., 2019a; Allen et al., 2019b). These published results, combined with the data produced in this study, suggest that fructose metabolism defects in ALS may contribute to the AGE influence on metabolic dysfunction, oxidative stress and inflammation and may be enhanced with aging.

Interestingly, we found that glycogen metabolism was negatively correlated with age in the ALS fibroblast cohort, which was not observed in control fibroblasts (Fig. 4). Glycogen can be stored in human diploid fibroblasts and accumulated under glucose starvation conditions (DiMauro and Mellman, 1973). In a recent paper focused on hippocampal metabolism in mice, an increase in glycogen metabolism enzymes including glycogen phosphorylase was observed in aged mice compared to young mice (Drulis-Fajdasz et al., 2018). We did not see this increase in the ALS iAstrocytes. Previously, CNS glycogen accumulation has been observed in SOD1 mouse models of ALS (Dodge et al., 2013) and evidence showed that glycogenolysis rather than glycogenesis is reduced in ALS (Li et al., 2019). In addition, previous data from our laboratory has shown that key glycogenolysis enzymes, glycogen phosphorylase and phosphoglucomutase enzymes were reduced in ALS patient derived astrocytes causing reduced glycogen metabolism (Allen et al., 2019b). A study in a FUS transgenic ALS mouse model found that glycogen synthase kinase‐3β (GSK‐3β), a kinase strongly associated with ALS, is activated and linked with deficiencies in ER-mitochondrial crosstalk (Stoica et al., 2016). Furthermore, GSK-3β is known to inhibit glycogen synthase resulting in reduced glycogenesis (Patel and Woodgett, 2017), which may be associated with reduced glycogen metabolism with age due to a lower level of glycogen availability in the cells. With this in mind, our data demonstrated a significant negative correlation of glycogen metabolism with age of onset and age of death in the FALS cohort. A similar trend was observed in the ALS cohort overall, but did not reach significance. Therefore, it is possible that changes in the levels of glycogen metabolising enzymes may occur with age in ALS, which could impact on the amount of energy produced from glycogen, and this in turn may influence disease parameters. We attempted to correlate the levels of these enzymes in control and ALS iAstrocyte cases with age (Fig. 4F-H). Although we saw reductions in GP with age, which was exacerbated in ALS iAstrocytes, we were limited by number of cell lines and no significant correlations were observed.

As glycogen metabolism correlated with ALS clinical parameters, we performed correlation analysis between the clinical parameters described and the other energy substrates in the phenotypic metabolic screen to investigate the possibility of similar effects. We found that two glycolytic intermediates, glucose-1-phosphate and D-fructose-6-phosphate were negatively correlated with disease progression in the FALS cohort (Fig. 5). Glycogen is converted to glucose-1-phosphate by GP, which is then converted to glucose-6-phosphate by PGM. Therefore, these data give weight to the idea that the glycogenolysis pathway may be altered in ALS, which could influence parameters of disease severity. At this point, it is unclear how D-fructose-6-phosphate metabolism is linked to disease progression rate especially as no correlation was observed with glucose-6-phosphate, which precedes D-fructose-6-phosphate in the glycolysis pathway (data not shown).

In contrast to the findings relating to glucose-1-phosphate and fructose-6-phosphate, we found a positive correlation of inosine as well as α-ketoglutaric acid metabolism with disease progression in the FALS cohort (Fig. 5). Previously, it has been shown that treatment with inosine significantly increased glycolytic flux and ATP production in both *C9orf72* and SALS iAstrocytes (Allen et al., 2019a). Our data add further weight to the hypothesis that increased inosine metabolism may be protective in ALS as inosine supplementation increased ALS fibroblast mitochondrial spare respiratory capacity and both glycolytic flux and capacity (Fig. 5E). A pilot study based on inosine administration in ALS patients increased urate levels in serum, where low levels had been documented in ALS patients (Nicholson et al., 2018; Paganoni et al., 2018). Increased inosine metabolism could enhance hypoxanthine levels via the purine degradation pathway in order to produce more uric acid (Dudzinska et al., 2010), which may influence antioxidant capacity (Nicholson et al., 2018). Oxidative stress is one of the hallmarks of ALS, so further evaluation of the effects of supplementary inosine on the disease course in ALS is warranted. Lastly, our data implied that patients live longer after disease onset when utilization of α-ketoglutaric acid as energy fuel is enhanced (Fig. 5). α-ketoglutaric acid has been associated with expanded lifespan in various species, including aged mice, worms and flies and increased levels have been found in plasma of elderly people (Asadi Shahmirzadi et al., 2020; Lawton et al., 2008; Mishur et al., 2016; Su et al., 2019). Specifically, in Drosophila, it was found that dietary supplementation of α-ketoglutaric acid increased lifespan via activation of AMPK signalling and enhanced autophagy (Su et al., 2019). Both were increased in the SOD1^G93A^ mouse model of ALS using an AMPK activator, causing a significant expansion in lifespan (Coughlan et al., 2015). In addition, defects in autophagy are associated with ALS toxic aggregates and more recently it was found *in vitro* that α-ketoglutarate inhibits starvation-induced autophagy, which is important function to ameliorate aggregation (Baracco et al., 2019). When we supplemented the ALS fibroblasts with α-ketoglutaric acid, we saw a significant increase in mitochondrial function (Fig. 5G-H). Taken together, these data suggest a possible protective role of α-ketoglutaric acid in ALS.

## Conclusions

Distinct from ALS, it is well established that the natural aging process affects metabolic function, whilst metabolic dysfunction is an early pathophysiological event in ALS. Here, we show that fibroblasts from ALS cases have a distinct metabolic profile, which is influenced by age. Correlation between age and catabolic metabolic substrates in fibroblasts, and validation in iAstrocytes, as well as supplementation assays showed increased metabolism of a distinct set of energy substrates with age in healthy individuals, which was not observed in ALS cases. Conversely, glycogen metabolism was negatively correlated with age in the ALS cohort compared to controls perhaps due to loss of glycogen metabolism enzymes with age. A key question from this study is how, or if at all, these metabolic changes are interconnected? Our previous work has shown that in *C9orf72* ALS iAstrocytes, multiple metabolic pathways are altered with similar results found in SALS iAstrocytes, leading to loss of metabolic flexibility (Allen et al., 2019b). These new data feed into that hypothesis and suggest that increased metabolic flexibility with age helps to counteract age related stress and maintain energy levels. In our previous studies, we showed reduction in glycogen, pyruvate, fructose and nucleoside metabolism in *C9orf72* iAstrocytes, which all feed carbon into glycolysis via multiple points. The present data are in alignment with our previously published data. Glycogen, xylitol, salicin, trehalose, fructose, turanose and lactic acid all feed carbon into glycolysis at specific points: glycogen via glucose-1-phosphate (Allen et al., 2019b), xylitol via the pentose phosphate pathway (Borgstrom et al., 2019), salicin via α-glucosidase action on glycogen (Dodge et al., 2013), fructose and turanose as previously described, trehalose by phosphotrehalase action to glucose (Zhang and DeBosch, 2020) and lactic acid by the action of lactate dehydrogenase to pyruvate (Ngo and Steyn, 2015). This suggests that modulation of glycolysis is not only neuroprotective (Manzo et al., 2019), but may protect against aging. Recently, an increase of autophagy in haematopoietic stem cells from aged mice was shown to contribute to healthier cell features such as long-term reconstitution, regeneration and higher cell numbers. The authors argued that shifting of the overall metabolism towards glycolysis contributed to increased cellular health (Ho et al., 2017). Our data suggest that supplementation with energy substrates such as inosine and pyruvate may be bioenergetically beneficial, the latter potentially via pyruvate dehydrogenase stimulation (Cunnane et al., 2020).

In addition to glycolysis convergence, we see TCA cycle convergence in our data set. Uridine and α-ketoglutaric acid both feed carbon into the TCA cycle, the former via acetyl co-A (Zhang et al., 2020). As α-ketoglutaric acid supplementation was also bioenergetically favourable, this suggests that as well as glycolytic energetic supplementation, TCA cycle supplementation may also increase energy output. This strategy has been used before with ketone body supplementation, which can directly provide the cell with acetyl co-A (Cunnane et al., 2020). Stimulation of both pathways in combination with trehalose supplementation to boost autophagy/mitophagy levels may represent an effective nutritional supplementation approach in ALS patients.

Taken together, the evidence we present in this report suggests potential loss of beneficial metabolic effects in ALS cases that may influence clinical parameters such as disease progression. Previous studies have shown that FALS as well as SALS patients exhibit similar clinical characteristics despite the diversity of symptoms, age of onset and disease duration (Al-Chalabi et al., 2016). However, there is no objective cut-off to distinguish between slow or late disease progression and early or late age of onset. Therefore, using patient specific metabolic profiles via metabotyping could be an important tool to distinguish these clinical characteristics, and metabolically stratify individuals with ALS. This study adds novel findings to existing knowledge linking metabolic alterations in healthy aging and in ALS. Moreover, our data add to the body of evidence that metabolism of specific metabolic substrates may influence clinical parameters in ALS patients such as disease progression.

## CRediT authorship contribution statement

**Margarita Gerou:** Methodology, Validation, Formal analysis, Investigation, Visualization, Writing – review & editing. **Benjamin Hall:** Methodology, Validation, Formal analysis, Investigation, Visualization, Writing – original draft. **Ryan Woof:** Methodology, Validation, Formal analysis, Investigation, Visualization, Writing – original draft. **Jessica Allsop:** Investigation, Validation. **Stephen J. Kolb:** Resources, Validation, Writing – review & editing. **Kathrin Meyer:** Resources, Validation, Writing – review & editing. **Pamela J. Shaw:** Conceptualization, Resources, Writing – original draft, Writing – review & editing, Funding acquisition. **Scott P. Allen:** Conceptualization, Methodology, Validation, Formal analysis, Investigation, Visualization, Writing – original draft, Resources, Supervision, Data curation, Supervision, Project administration, Visualization.

## Conflict of interest

The authors can state there is no conflict of interest associated with this work.

## Appendix Supplementary materials

Image, application 1

Image, application 2

Image, application 2

Image, application 2

Image, application 2
