# Supplementary material for: Amyotrophic lateral sclerosis alters the metabolic aging profile in patient derived fibroblasts
Source: Neurobiol Aging. 2021 Sep;105:64–77. doi: 10.1016/j.neurobiolaging.2021.04.013 (PMC8346650; doi:10.1016/j.neurobiolaging.2021.04.013)

**Supplementary Figure 1. No correlation between susceptibility to starvation induced toxicity and age was observed in fibroblasts**

Data presented as mean with standard deviation of at least three biological repeats per cell line. Pearson’s correlation analysis showed no difference in either the control group (n=15, black) or ALS group (n=21, pink) with the p value set to ≤0.05.

**
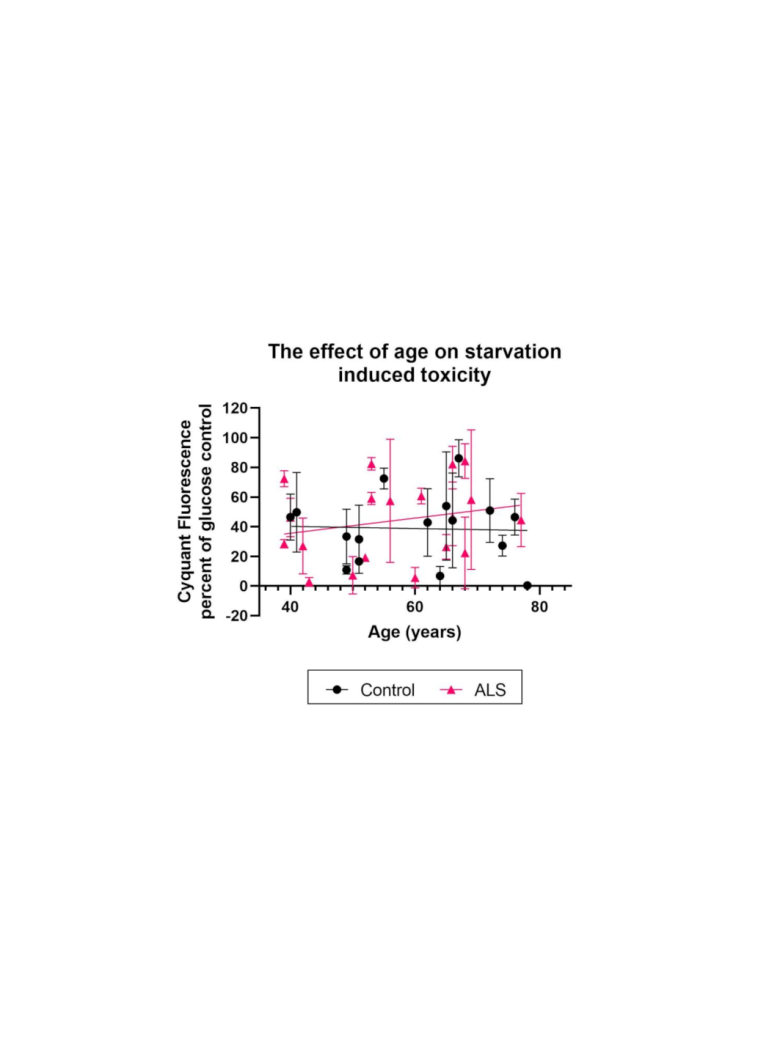
**

**Supplementary Figure 2. Western blot analysis of glycogen phosphorylase (GP) and phosphoglucomutase (PGM) levels in iAstrocytes.**

C9-ALS = *C9orf72* samples, SALS = sporadic samples. Age (years) = age at biopsy.


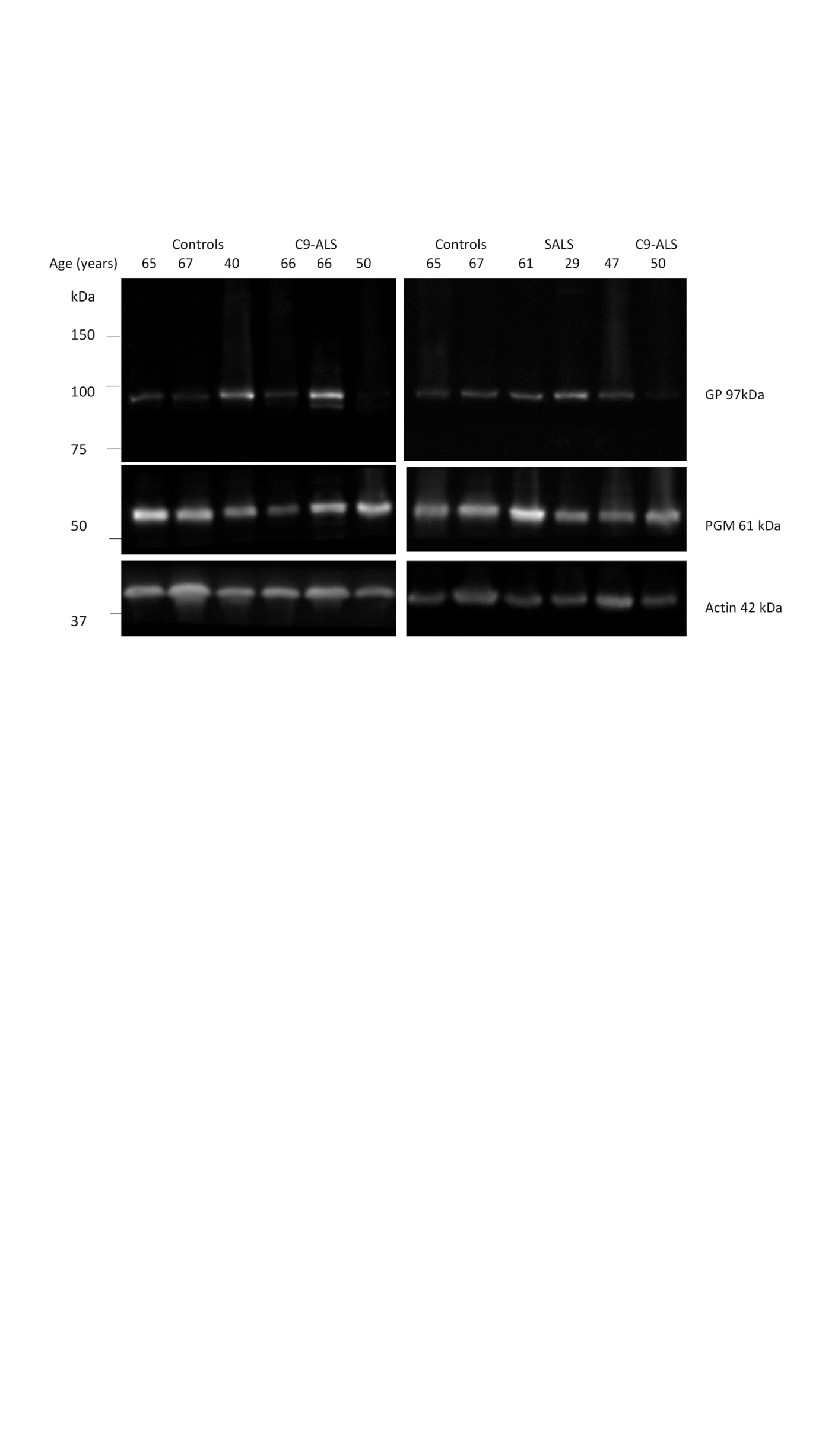

Supplement: Supplementary file 1 [file mmc1.docx]
